# Glucosyl hesperidin exhibits more potent anxiolytic activity than hesperidin accompanied by the attenuation of noradrenaline induction in a zebrafish model

**DOI:** 10.3389/fphar.2023.1213252

**Published:** 2023-08-17

**Authors:** Takumi Nishida, Chihoko Horita, Mikiya Imagawa, Momoka Hibarino, Sayaka Tateno, Yurina Kubo, Momoko Kawabe, Naoki Morishita, Shin Endo, Kazuhiro Shiozaki

**Affiliations:** ^1^ Department of Food Life Sciences, Faculty of Fisheries, Kagoshima University, Kagoshima, Japan; ^2^ Course of Biological Science and Technology, The United Graduate School of Agricultural Sciences, Kagoshima University, Kagoshima, Japan; ^3^ R&D Division, Hayashibara Co., Ltd., Okayama, Japan

**Keywords:** anxiety, behavior, glucosyl hesperidin, hesperidin, noradrenaline, zebrafish

## Abstract

Anxiety is a symptom of various mental disorders, including depression. Severe anxiety can significantly affect the quality of life. Hesperidin (Hes), a flavonoid found in the peel of citrus fruits, reportedly has various functional properties, one of which is its ability to relieve acute and chronic stress. However, Hes is insoluble in water, resulting in a low absorption rate in the body and low bioavailability. Glucosyl hesperidin (GHes) is produced by adding one glucose molecule to hesperidin. Its water solubility is significantly higher than that of Hes, which is expected to improve its absorption into the body and enhance its effects. However, its efficacy in alleviating anxiety has not yet been investigated. Therefore, in this study, the anxiolytic effects of GHes were examined in a zebrafish model of anxiety. Long-term administration of diets supplemented with GHes did not cause any toxicity in the zebrafish. In the novel tank test, zebrafish in the control condition exhibited an anxious behavior called freezing, which was significantly suppressed in GHes-fed zebrafish. In the black-white preference test, which also induces visual stress, GHes-fed zebrafish showed significantly increased swimming time in the white side area. Furthermore, in tactile (low water-level stress) and olfactory-mediated stress (alarm substance administration test) tests, GHes suppressed anxious behavior, and these effects were stronger than those of Hes. Increased noradrenaline levels in the brain generally cause freezing; however, in zebrafish treated with GHes, the amount of noradrenaline after stress was lower than that in the control group. Activation of c-fos/ERK/Th, which is upstream of the noradrenaline synthesis pathway, was also suppressed, while activation of the CREB/BDNF system, which is vital for neuroprotective effects, was significantly increased. These results indicate that GHes has a more potent anxiolytic effect than Hes *in vivo*, which may have potential applications in drug discovery and functional food development.

## 1 Introduction

Flavonoids are secondary metabolites that are abundant in plants, fruits, and seeds, and are responsible for color, fragrance, and flavor characteristics (Dias et al., 2021). Flavonoids possess various physiological functions, including anti-oxidant activity, regulation of cell growth and differentiation, inhibition of inflammation, suppression of bacterial infection, and reduced risk of human diseases (Yoshinaga et al., 2016; Shinyoshi et al., 2017; Dias et al., 2021; Domaszewska-Szostek et al., 2021; Silva et al., 2021; Deng et al., 2022).

Hesperidin (hesperetin 7-rutinoside, Hes) is a flavanone glycoside comprising hesperetin and rutinose. Hesperidin is abundant not only in citrus fruits, such as lemon (*Citrus Limon*), sweet oranges (*Citrus sinensis*), bitter orange (*Citrus aurantium*), citron (*Citrus medica*), clementines (*Citrus clementina*), and mandarins (*Citrus reticulata*) (Wdowiak et al., 2022) but also in other fruits and seaweeds (Jeon et al., 2014; Grina et al., 2020).

The health benefits of Hes have been discussed for various diseases. For example, Hes suppresses cancer cell growth by inducing apoptosis through the PI3/AKT pathway (Aggarwal et al., 2020). Hes attenuates nitric oxide deficiency-induced cardiovascular remodeling by suppressing the expression of TGF-β1 and matrix metalloproteinase proteins, MMP-2 and MMP-9 (Maneesai et al., 2018). Hes decreases diabetic nephropathy induction by modulating TGF-β1 and oxidative DNA damage (Kandemir et al., 2018).

The anxiolytic and antidepressant-like activities of Hes have been recently reported. Anxiety is a typical symptom of depression and other psychiatric disorders that affect many patients worldwide. Hes suppressed anxious behavior in Parkinson’s disease model mice in the Elevated Plus-Maze Test (EPMT) and splash test (Antunes et al., 2020). Hes exhibited antidepressant-like effects on the EMPT, forced swimming test, and open field test in streptozotocin-induced diabetic rats (Zhu et al., 2020). Hes improved depression-like behaviors in rats after exposure to a single prolonged stress (post-traumatic stress model), accompanied by a decrease in freezing behavior (Lee et al., 2021). Hes is also the main component of Chin-pi, a Chinese medicinal herb originating from citrus peels that shows anxiolytic activity in rodents (Ito et al., 2013).

Although numerous physiological functions have been reported for Hes, significant metabolic problems are associated with its poor bioavailability, similar to many other flavonoids. In general, Hes is hydrolyzed to hesperetin aglycone by the intestinal microbiota. The absorbed hesperetin is metabolized by UDP-glucuronosyl transferases and sulfotransferases in the colon, small intestine, and liver at the 3′- and 7-position (Boonpawa et al., 2017). Hesperetin and hesperetin glucuronide can traverse the blood-brain barrier (BBB) *in vitro* (Youdim et al., 2003). The expression of the biological functions of orally administered Hes is reliant on this metabolic pathway. However, the water solubility of Hes is low (0.002 g/100 g water) and has low absorption efficiency in the intestine, resulting in insufficient bioactivity of Hes metabolites.

Glucosyl hesperidin (GHes) is a conjugate of monoglucose with Hes and is produced using Cyclodextrin Glucanotransferase (CGTase) originating from *Bacillus* species that can conjugate monoglucose to Hes (Chen et al., 2022). The water solubility of GHes is approximately 10,000 times higher than that of Hes (Yamada et al., 2006). As expected, the serum hesperetin concentration increased more rapidly in rats administered GHes than in those administered Hes (Yamada et al., 2006). The area under the concentration-time curve for hesperetin in the sera of rats administered GHes was approximately 3.7-fold greater than that in rats administered Hes (Yamada et al., 2006). The physiological functions of GHes include the inhibition of influenza viral sialidase activity (Saha et al., 2009), clinical trials for preventing obesity (Yoshitomi et al., 2021), inhibition of selenite-induced cataract formation (Nakazawa et al., 2020), and inhibition of gravity-induced lower-leg swelling (Nishimura et al., 2021). These reports suggest that the anxiolytic activity of GHes may be greater than that of Hes. However, despite the high water solubility of GHes, no differences in blood pressure reduction or other effects in hypertensive rats were reported (Ohtsuki et al., 2002; Ikemura et al., 2012). Furthermore, the effects of GHes on anxiety behaviors have not yet been investigated.

To investigate the effectiveness of GHes in human health research, the current study aimed to evaluate the anxiolytic activity of GHes compared with Hes. In this study, we used zebrafish as a model of anxiety. Zebrafish are small fish belonging to the Cypriniformes family and are officially recognized by the NIH as the third most commonly used laboratory animal after mice and rats (Zhao et al., 2018). Zebrafish are relatively inexpensive to maintain and require less space than rodents. We also have access to the whole genome information of zebrafish, and approximately 70% of human genes are conserved in zebrafish. Methods to study anxiety, such as open field tanks, black-and-white preference, and t-maze tests, have been developed in zebrafish as well as in mice (Shiozaki et al., 2020). Zebrafish are suitable for evaluating antidepressant drugs or natural compounds. In this study, we performed a novel tank test, black-white preference test, and acute stress induction by low water level and alarm substance to determine the effects of GHes on anxiety behavior in zebrafish. We also investigated the underlying mechanism by which GHes suppressed anxiety.

## 2 Materials and methods

### 2.1 Zebrafish

The RIKEN WT (RW) zebrafish strain was supplied by the Center for Brain Science, Institute of Physical and Chemical Research (RIKEN, Saitama, Japan). Adult zebrafish (6–12 months old) were raised in a 2-L water aquarium with a 14/10 h light/dark cycle at 28°C. They were fed live brine shrimp and a commercial diet (Otohime B2; Marubeni Nisshin Feed Co., Ltd., Tokyo, Japan) twice daily. We set the ratio of the number of male and female zebrafish used in our experiments to 1:1. All the animal experimental protocols were approved by the Kagoshima University Committee (ethics protocol No. F23001).

### 2.2 Administration of GHes and hes

GHes (97% purity, Hayashibara Co., Ltd, Okayama, Japan) or Hes (95% purity, LKT LAB, MN, United States) added diet was prepared as follows: GHes or Hes was mixed with pulverized commercial diet to a 1% concentration, then make it freeze-dried and pellet (0.6–1.0 mm). Under the conditions of this study, feeding 1% Hes to zebrafish is roughly equivalent to 200 mg Hes/day/Kg body weight. Similar Hes concentrations have been used in several studies (Ahmadi et al., 2008; Fu et al., 2019). A control diet was prepared using the above methods without GHes or Hes. The diets were preserved at −20°C during the administration period. The administration was conducted in 2-L tanks at 28°C. The zebrafish were separated into 2-L tanks and fed a commercial diet until the experimental diet was administered. Fish were fed to apparent satiation twice a day Feeding was carried out by repeatedly feeding the zebrafish a small amount of experimental diet until they stopped eating. Food intake was expressed as the total diet ingested in each tank/fish number.

### 2.3 Evaluation of behavior

#### 2.3.1 Motility test

A motility test was conducted using zebrafish-fed control, Hes, or GHes for 52 days. A fish was introduced into the white tank (21.6 cm wide, 22.8 cm long, 12 cm depth), habituated for 15 min, and the swimming behaviors of the zebrafish were recorded using a video camera (HDR-CX430, Sony, Tokyo, Japan) for 5 min. Swimming was automatically recorded and tracked using Move-tr/2D software (Library, Tokyo, Japan). The total distance traveled, and swimming velocity were analyzed.

#### 2.3.2 Novel tank test

The novel tank test was carried out using zebrafish that were fed control, Hes, or GHes diets for 31 days. Fish were introduced into the center of a bright white tank (21.6 cm wide, 22.8 cm long, 12 cm depth), and their swimming behavior was recorded using a video camera for 5 min. Swimming was automatically tracked using Move-tr/2D software. The total freezing time, freezing frequency, and distance traveled were analyzed.

#### 2.3.3 Black-white preference test

The black-white preference test was performed with slight modifications using zebrafish that were fed control, Hes, or GHes diets for 21 days (Ikeda et al., 2021). A fish was introduced into the black section of the tank (23 cm wide, 13 cm long, and 6 cm height), which was divided into half black-and-white sections, and the swimming behavior of the zebrafish was recorded using a video camera for 20 min. Swimming was automatically tracked using Move-tr/2D software. The total swimming time in the black area, total time in the white area, and total frequency of invasion into the white area were analyzed.

#### 2.3.4 Low water level stress test

The low water level test was conducted with slight modifications using zebrafish that were fed either control, Hes, or GHes diets for 14 days (Piato et al., 2011). Low water level stress was defined as the zebrafish dorsal being out of the water surface. The fish were exposed to low water level stress for 2 min, then introduced into a white tank (21.6 cm wide, 22.8 cm long, 12 cm depth), and their swimming behavior was recorded using a video camera for 10 min. Five minutes of swimming were automatically tracked using Move-tr/2D. The total freezing time and frequency over 10 min were analyzed.

#### 2.3.5 Alarm substance exposure test

The alarm substance exposure test was conducted using zebrafish that were fed with control, Hes, or GHes diets for 7 days. The alarm substance was prepared as described previously (Speedie and Gerlai, 2008). Briefly, zebrafish scales were peeled with a scalpel and finely crushed in cold PBS. The homogenate was centrifuged, and its supernatant was stored at −80°C until use. A fish was introduced into the transparent tank (18.5 cm, 10.9 cm, 11 cm height) with the alarm substance (equivalent to the amount derived from 0.03 fish/L), and the swimming behavior of the zebrafish was recorded using a video camera for 10 min. Swimming for 3 min was automatically tracked using Move-tr/2D. The total freezing time was analyzed for 10 min.

### 2.4 Real-time PCR

The mRNA expression levels of each gene were analyzed using cDNAs from the zebrafish brain using a Step One Real-Time System (Thermo Fisher Scientific, MA). Zebrafish brains were removed after euthanasia with 0.1% tricaine. Tricaine has been used in many studies on anxiety in fish and has been reported to have no effect on anxiety or stress-related behaviors (Nordgreen et al., 2014). Total RNA was extracted from the zebrafish brains using Sepasol-RNA I Super G solution (Nacalai Tesque, Kyoto, Japan), and cDNA synthesis was performed using ReverTra Ace qPCR RT Master Mix with gDNA Remover (TOYOBO, Osaka, Japan). Real-time PCR was conducted using KOD SYBR qPCR Mix or THUNDERBIRD qPCR Mix (TOYOBO). The specific primers used for PCR are listed in Supplementary Table S1. The expression level of *actb* mRNA was used as an internal standard to compensate for the quality and quantity of mRNA in each sample. Primers were designed by using NCBI Primer-BLAST (https://www.ncbi.nlm.nih.gov/tools/primer-blast/).

### 2.5 Immunoblotting

Zebrafish brains were homogenized in lysis buffer (50 mM 4-(2-hydroxyethyl)-1-piperazineethanesulfonic acid (pH 7.4), 150 mM NaCl, 1% NP-40, 2 mM ethylenediaminetetraacetic acid, 10 µg/mL leupeptin, 10 mM sodium fluoride, 2 mM sodium orthovanadate, 0.25% sodium deoxycholate, and 0.2 mM phenylmethyl sulfonyl fluoride).

The lysates were separated using 10% acrylamide gel electrophoresis and transferred onto polyvinylidene difluoride (PVDF) membranes. The blocking of the membrane was performed with 1% bovine serum albumin (BSA) in PBS containing 0.1% Tween 20 (PBST) and incubated with primary antibodies against anti-phospho ERK and anti-ERK (polyclonal, 1/1,000 dilution; Cell Signaling Technology, MA, United States), anti-β-actin (clone 2D4H5; 1/1,000 diluted; Proteintech, IL, United States), anti-CREB (clone, D-12;1/500 dilution; Santa Cruz Biotechnology, TX, United States), and anti-TH (polyclonal, 1/1,000 dilution; GeneTex, CA, United States), followed by incubation with HRP-conjugated secondary antibodies. Bands were detected using the EzWestLumi Plus chemiluminescence reagent (ATTO, Tokyo, Japan) with ChemiDoc Touch Plus (Bio-Rad Laboratories, CA, United States), and densitometric analysis was conducted using Image Lab Touch software (Bio-Rad).

### 2.6 Determination of monoamines

Noradrenaline, serotonin, and dopamine levels were determined as described previously, with slight modifications (Kawano et al., 2020). Briefly, fresh zebrafish brains were homogenized in 0.2 M perchloric acid containing 0.1 mM EDTA. After centrifugation at 12,000 × *g*, the supernatant was mixed with 0.2 M sodium acetate and analyzed using HPLC. The HPLC system consisted of a pump (JASCO PU-4180, JASCO, Tokyo, Japan), an autosampler (JASCO AS-4550), a column oven (JASCO CO-4061), and an electrochemical detector (ECD-700, EiCOM, Kyoto, Japan) with a graphite carbon working electrode and an Ag/AgCl reference electrode. The ECD potential was set at +750 mV for the working electrode. The mobile phase was an acetate-citrate buffer (pH 3.5) containing 0.053 M citric acid, 0.047 M sodium acetate, 5 mg/L EDTA, 195 mg/L sodium octyl sulfonate, and 17% methanol (v/v). The mobile phase was delivered at a flow rate of 0.5 mL/min to a stainless steel column (Eicompack SC-5ODS, 3 mm φ × 150 mm; EiCOM).

### 2.7 Data analysis

Results are presented as mean ± standard deviation of the mean. Normality test and Group size used in this study were estimated using IBM SPSS statistics software (Armonk, NY). In the two groups, data were compared using a *t*-test. In the three groups, data were compared using a one-way analysis of variance (ANOVA) followed by Tukey’s multiple comparison test.

## 3 Results

### 3.1 GHes attenuated the anxiety induced via visual stress

**FIGURE 1:**
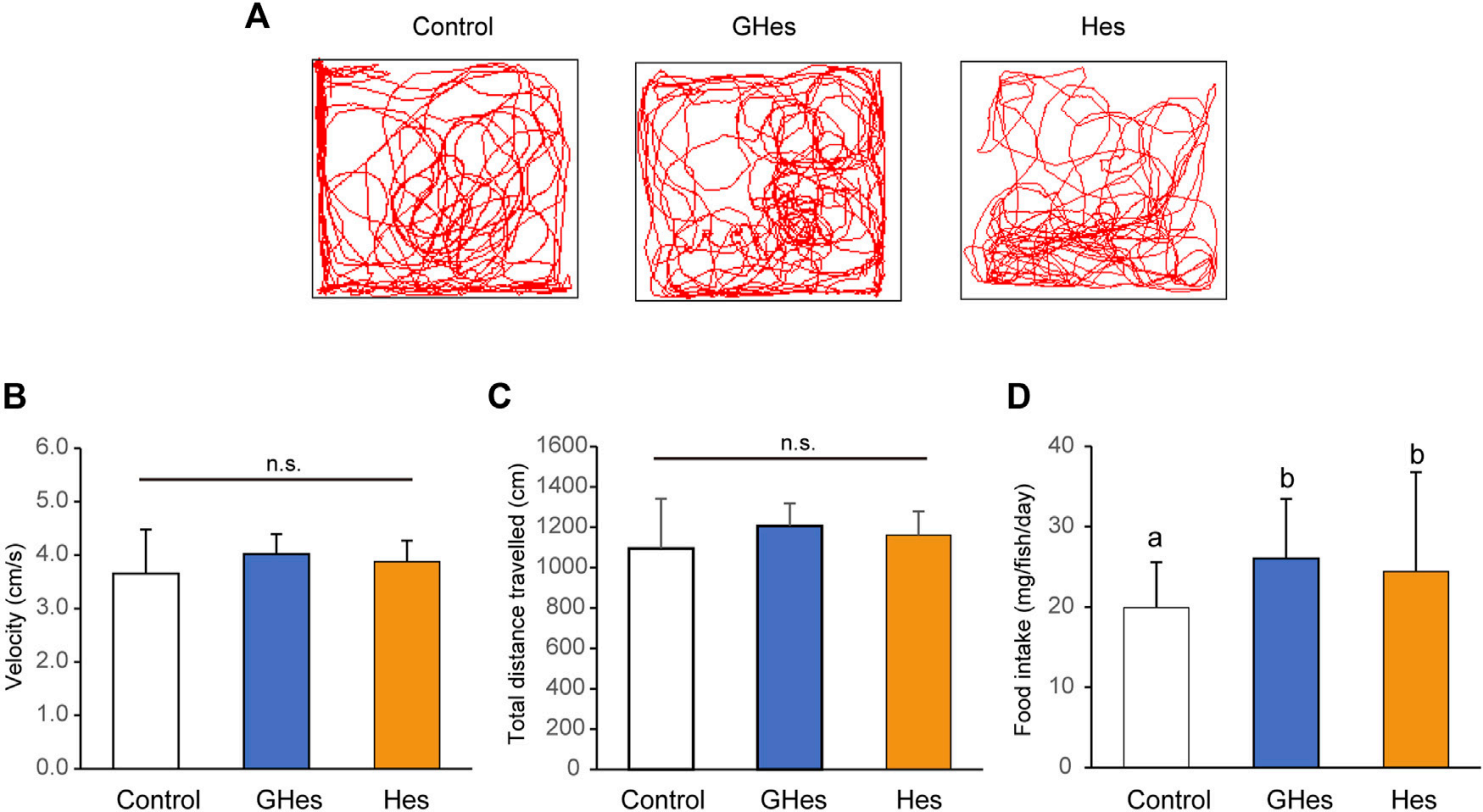
Effect of GHes and Hes on non-stress zebrafish behavior. Zebrafish were fed a control diet, GHes-diet, or Hes-diet for 52 d. **(A)** Tracking in a familiar tank for 5 min. **(B)** Swimming velocity. **(C)** Swimming distance. **(D)** Average food intake per day and fish. Results are shown as means ± standard deviation. *n* = 6. n.s not significant. Columns with the same letter are not statistically different, and *vice versa*.

Before evaluating the anxiolytic activity of GHes, the effects of GHes on non-stressed zebrafish were compared with those of the control and Hes. Zebrafish that were fed GHes or Hes did not differ from the controls in swimming trajectory, swimming speed, or swimming distance (Figures 1A–C). GHes and Hes slightly but significantly enhanced the daily food intake in zebrafish compared to the control (*p* < 0.01 in GHes and Hes vs control) (*F* = 11.609, *p* < 0.0001 in One-way ANOVA, Figure 1D).

**FIGURE 2:**
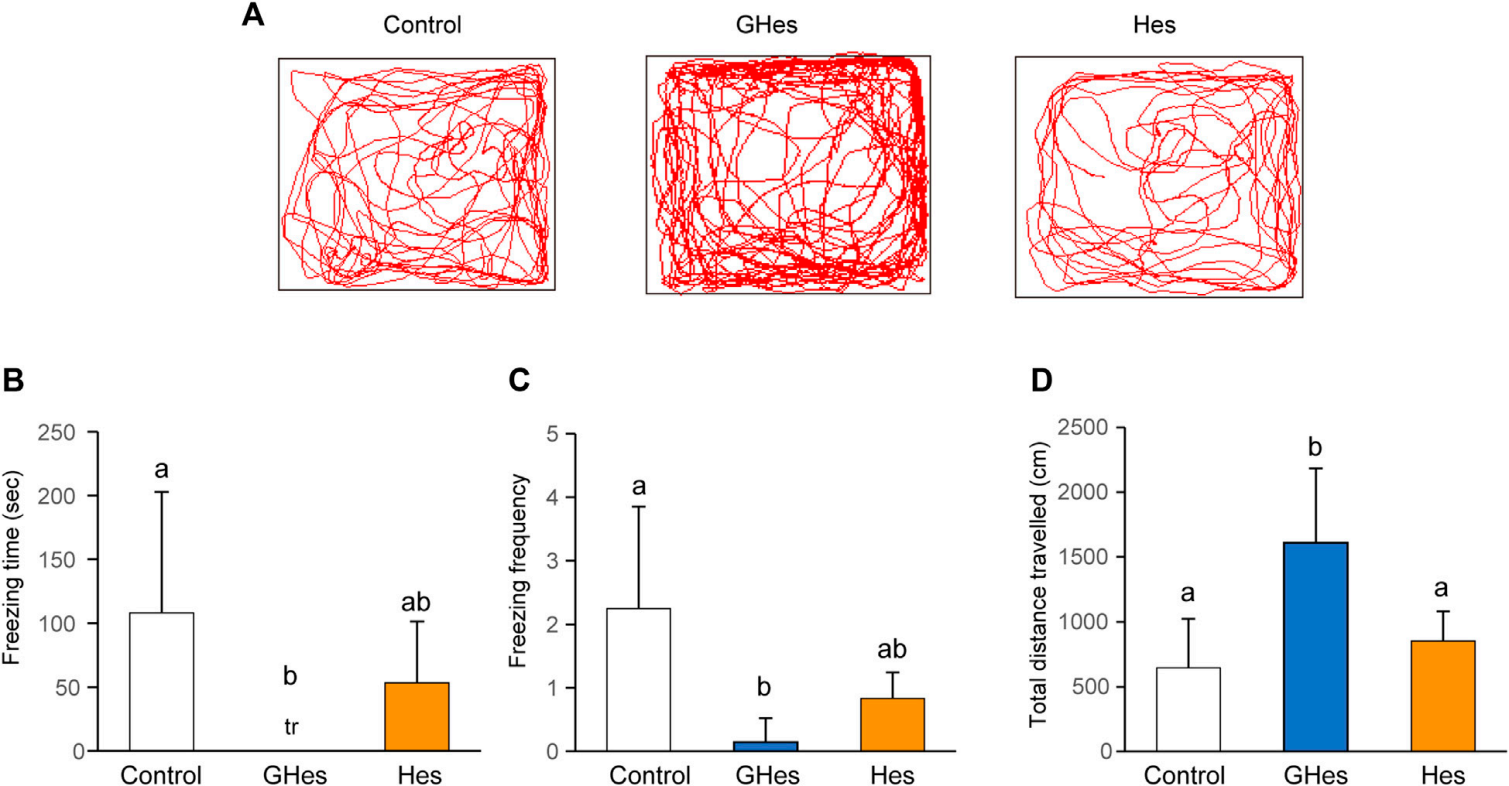
Effect of GHes and Hes on zebrafish behavior in novel tank test. Zebrafish were fed control-, GHes-, or Hes-diet for 31 days and subsequently used for novel tank test, **(A)** Tracking of zebrafish swimming with control, GHes, or Hes-diet. **(B)** Freezing time during the 10 min observation. **(C)** Frequency of freezing. **(D)** Total distance traveled. *n* = 7. Results are shown as means ± standard deviation. n.s,. not significant. tr, trace. Columns with the same letter are not statistically different, and *vice versa*.

To evaluate the effect of GHes on anxiety behavior in zebrafish, a novel tank test, the visual stress test, was carried out. Under novel conditions, control-fed zebrafish exhibited freezing behavior accompanied by a drastic change in the swimming track (Figure 2A). However, GHes-fed zebrafish drastically suppressed freezing time (*p* < 0.05, vs control) (*F* = 4.489, *p* = 0.024 in one-way ANOVA, Figure 2B) and the frequency of freezing (*p* < 0.01 vs control) (*F* = 7.899, *p* = 0.0026 in one-way ANOVA, Figure 2C), and its swimming track was similar to that of non-stressed zebrafish, as shown in Figure 1A. According to the decreased anxiety behavior, the total distance traveled in the GHes was significantly elevated due to the increase in normal swimming (*p* < 0.01 vs control) (*F* = 11.691, *p* = 0.00039 in one-way ANOVA, Figures 2A,D). In contrast, Hes did not exhibit anxiolytic activity compared to the control (Figure 2).

**FIGURE 3:**
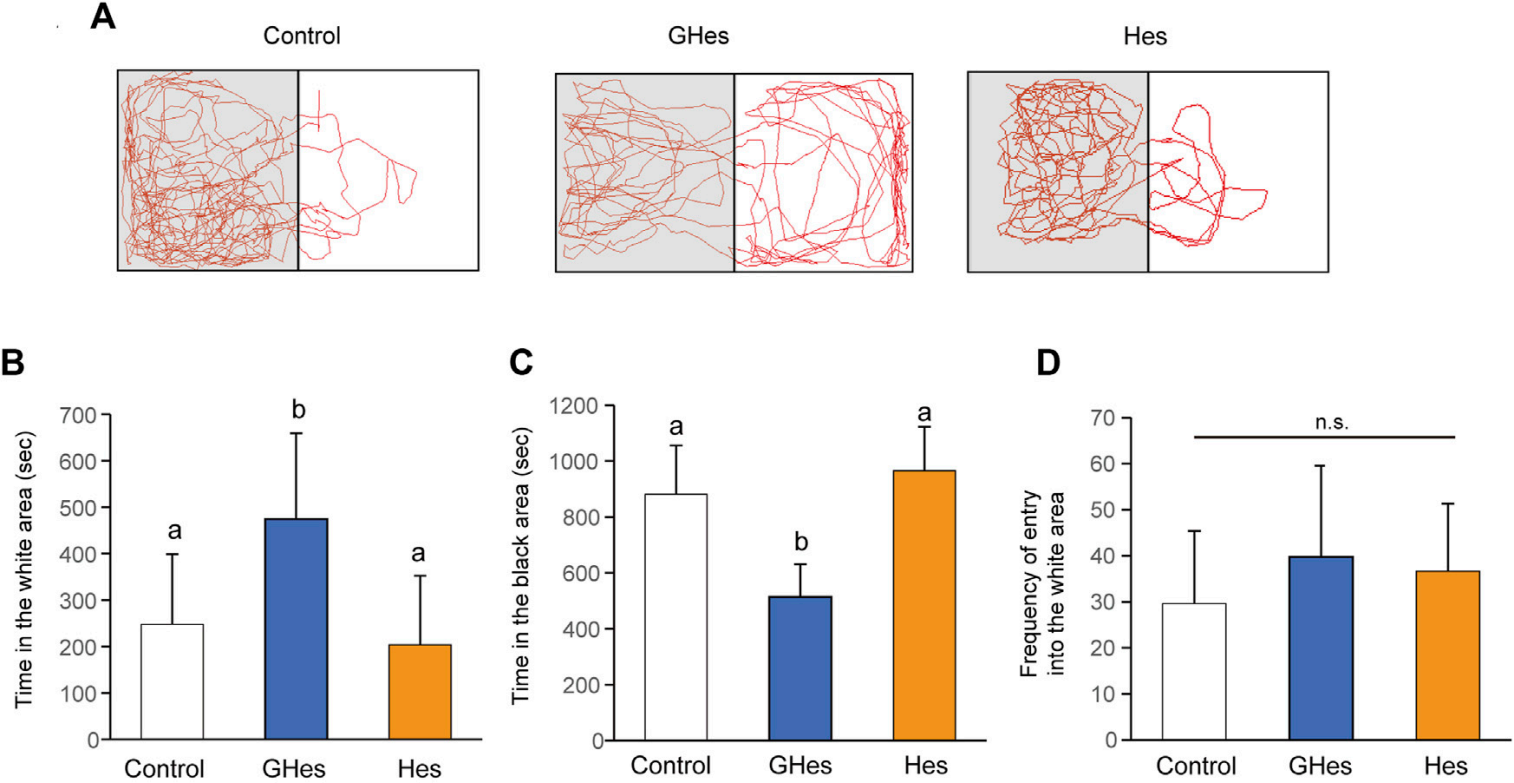
Effect of GHes and Hes on zebrafish behavior in the black-white preference test. Zebrafish were fed control, GHes, or Hes diet for 21 days and subjected to the black-white preference test. **(A)** Tracking zebrafish behavior. The gray and white colors in this picture indicate the black and white areas in the test tank, respectively. **(B)** Total swimming time in the white area. **(C)** Total swimming time in the black area. **(D)** Frequency of invasion of the white area. Results are shown as means ± standard deviation. *n* = 10. n.s., not significant. Columns with the same letter are not statistically different, and *vice versa*.

A black-white preference test was carried out to confirm anxiety suppression by GHes toward visually induced stress. Fish tended to prefer black areas because of their instinct to hide and feel insecure about white areas. However, when anxiety decreases, they swim to the white side in interest-seeking behaviors (Ikeda et al., 2021). As expected, control and Hes diet-fed zebrafish swam mainly in the black areas (Figure 3A). Control fish swam for over 800 s in the black area and 260 s in the white area (Figures 3B,C). In contrast, GHes-fed zebrafish exhibited different swimming patterns compared to the control fish (Figure 3A). GHes-fed fish significantly increased swimming time in the white area (*p* < 0.01 vs control) (*F* = 6.751, *p* = 0.0045 in one-way ANOVA, Figure 3B) and decreased swimming time in the black area (*p* < 0.01 vs control) (*F* = 19.709, *p* < 0.001 in one-way ANOVA, Figure 3C). The frequency of invasion into the white area did not differ among the control, GHes, and Hes groups, indicating that swimming time per invasion was increased on the white side by GHes treatment. These results suggest that GHes attenuates the anxiety caused by visual stress.

### 3.2 GHes decreased anxiety behavior induced by low water level stress

**FIGURE 4:**
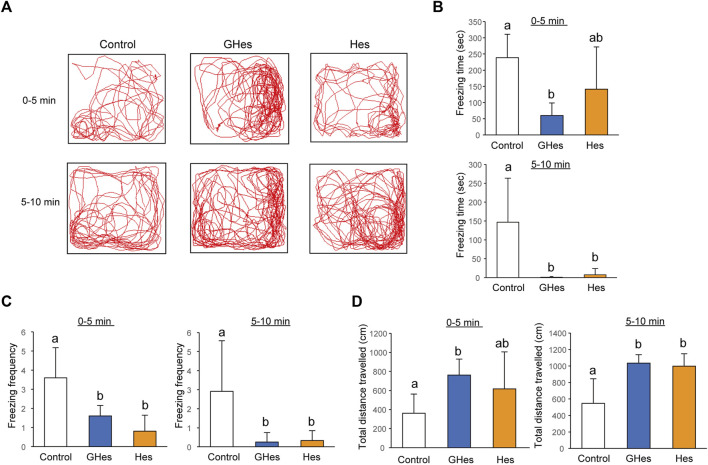
Effects of GHes and Hes on zebrafish behavior under the low water level-induced stress. Zebrafish were fed control, GHes, or Hes diet for 14 days and subjected to a low-water level stress test. **(A)** Zebrafish were tracked after stress induction. Upper panel: 0–5 min after stress induction. Lower panel: 5–10 min after stress induction. **(B)** Total Freezing time. **(C)** Freezing frequency. **(D)** Total distance traveled for 0–5 and 5–10 min. Results are shown as means ± standard deviation. *n* = 5. n.s., not significant. Columns with the same letter are not statistically different, and *vice versa*.

As this study revealed that GHes reduced anxiety behaviors induced by visual stress, we evaluated the effects of GHes on other stress-induced anxiety behaviors. Low water levels are stimuli recognized by the zebrafish body and are known to induce anxiety (Piato et al., 2011). This test examined two time periods: 0–5 min and 5–10 min. Control-fed fish exhibited drastic freezing behavior during both periods (Figures 4A,B). GHes significantly decreased freezing time in both periods compared to the control (*p* < 0.01, *p* < 0.05 vs control at 0–5 and 5–10 min, respectively) (*F* = 6.61, 6.208, and *p* = 0.007 and *p* = 0.0089, in 0–5 and 5–10 min, respectively, in one-way ANOVA, Figures 4B,C). Hes did not exhibit a reduction in freezing time from 0 to 5 min (Figures 4A,B) but showed a significant decrease from 5 to 10 min (*p* < 0.05; Figures 4A,C), indicating that Hes allowed zebrafish to recover faster from freezing than the control, but less than GHes. According to the reduction in freezing time, the total distance traveled in GHes-fed zebrafish in 0–5 min (*p* < 0.05, vs control) (*F* = 4.100, *p* = 0.034 in one-way ANOVA, Figure 4D) and that in GHes and Hes-fed fish in 5–10 min (*p* < 0.01, vs control) (*F* = 10.167, *p* = 0.0011 in one-way ANOVA, Figure 4D). These results suggest that GHes and Hes attenuate the acute stress induced by low water stimulation and that GHes is more anxiolytic than Hes.

### 3.3 GHes decreased anxiety behavior induced by alarm substance exposure

**FIGURE 5:**
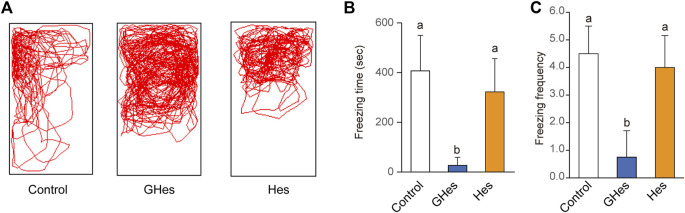
Effects of GHes and Hes on zebrafish behavior under the alarm substance-induced stress. Zebrafish were fed control, GHes, or Hes diet for 7 days and subjected to the alarm substance-induced stress test. **(A)** Zebrafish were tracked after stress induction. **(B)** Total freezing time and **(C)** freezing frequency. Results are shown as means ± standard deviation. *n* = 5. n.s., not significant. Columns with the same letter are not statistically different, and *vice versa*.

Alarm substances are secreted from damaged zebrafish skin to alert other fish to danger; their primary component is hypoxanthine 3-N-oxide (Parra et al., 2009). Fish recognize compounds based on their sense of smell and danger. Exposure to alarming substances induces anxiety in zebrafish. Control-fed fish exhibited drastic freezing and tended to swim in one corner (Figure 5). GHes treatment drastically suppressed freezing behavior time (*p* < 0.01, vs control) (*F* = 11.937, *p* = 0.0022 in one-way ANOVA, Figures 5A,B) and the frequency of freezing (*p* < 0.05, vs control) (*F* = 15.308, *p* = 0.0013, Figure 5C) by the alarm substance, similar to other acute stress experiments in this study. Hes did not exhibit decreased freezing behavior (Figure 5).

**FIGURE 6:**
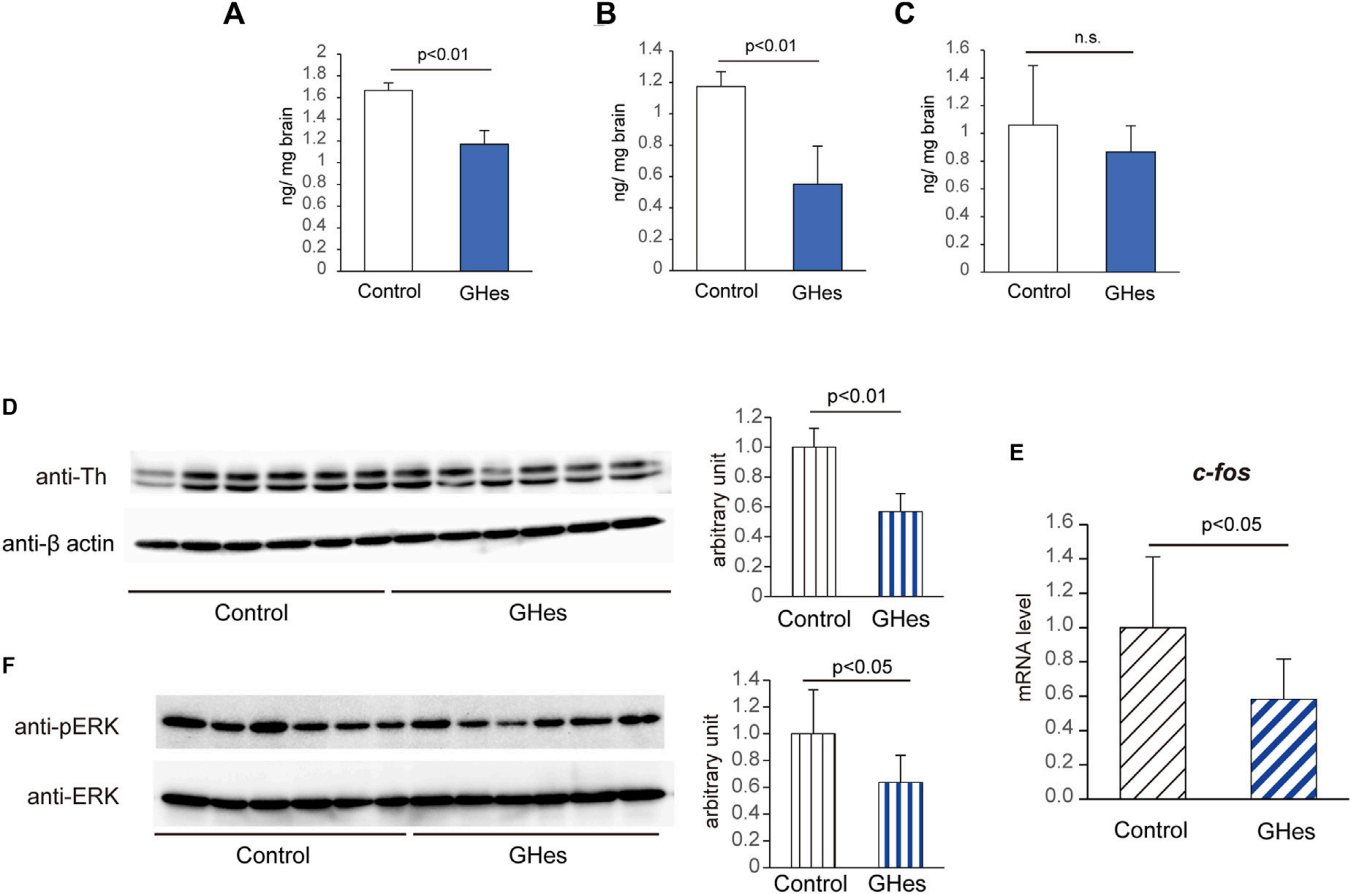
Alteration of the noradrenaline pathway in GHes-fed zebrafish. Control- or GHes-fed zebrafish (7 days) were exposed to alarm substance stress. **(A–C)** The fish brains were excised 5 min after stress, and the contents of noradrenaline **(A)**, dopamine **(B)**, and serotonin **(C)** in the brain were estimated using HPLC. **(D and F)** Fish brains were excised 15 min after stress. Brain lysates were subjected to Western blotting with anti-Th **(D)** and anti-phospho-ERK **(F)** antibodies. Actin and ERK were used as internal controls. Quantitative analyses of the intensities of the protein bands were conducted, and the results are presented as Th/β-actin and p-ERK/ERK. *n* = 8. **(E)** Fish brains were excised 15 min after stress induction. *c-fos* mRNA levels in the zebrafish brains were assessed using real-time PCR. Each level of gene expression in GHes-fed zebrafish was relative to that in control. *n* = 10. n.s., not significant. Results are shown as means ± standard deviation.

As noradrenaline (NA) is involved in the induction of freezing via the ERK/AP-1 pathway (Miller et al., 2010), NA content was expected to be altered in GHes-fed fish under alarm substance stimulation. As expected, the NA content in the whole brain was significantly suppressed in GHes-fed zebrafish compared to the control (*p* < 0.01, Figure 6A). Dopamine, a precursor of NA, was also significantly decreased in GHes-treated fish (*p* < 0.01, Figure 6B), while the level of serotonin, a regulator of NA neurons, was not altered (Figure 6C).

Tyrosine hydroxylase 1 (Th1) catalyzes the conversion of the amino acid L-tyrosine to L-3,4-dihydroxyphenylalanine (L-DOPA), which is responsible for catecholamine synthesis, including noradrenaline. To understand the mechanism by which GHes reduces freezing behavior in zebrafish, we analyzed alterations in the ERK/AP-1/TH1 pathway. As expected, GHes suppressed the expression of Th1 polypeptides compared to that in control (*p* < 0.01, Figure 6D and Supplementary Figure S1), which coincided with a decrease in NA content in GHes-fed zebrafish. The gene expression of *c-fos*, a marker of activated neurons and a component of AP-1, was also suppressed in the GHes-fed zebrafish (*p* < 0.05, Figure 6E and Supplementary Figure S1). The phosphorylation of ERK, upstream of AP-1, was also downregulated by GHes (*p* < 0.05, Figure 6F and Supplementary Figure S1).

**FIGURE 7:**
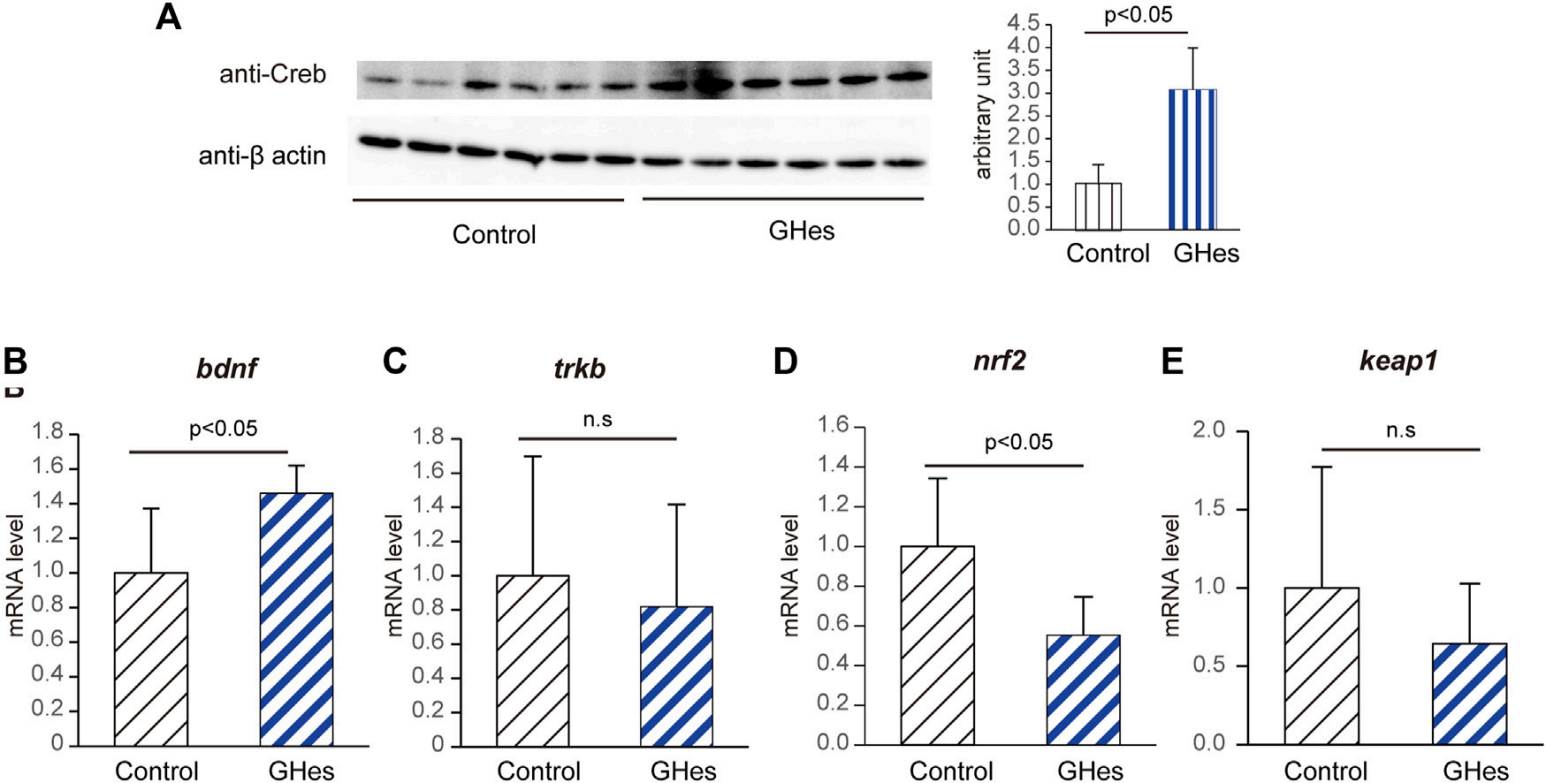
Alteration of CREB/BDNF pathway in GHes-fed zebrafish. Control- or GHes-fed zebrafish (7 days) were exposed to alarm substance stress. The brains were excised 15 min after the stress exposure. **(A)** Brain lysates were subjected to Western blotting with an anti-Creb antibody. *β*-actin was used as an internal control. Quantitative analyses of the intensities of the protein bands were conducted, and the results are presented as Creb/β-actin. *n* = 5. **(B–E)** Fish brains were excised 15 min after exposure to stress. **(B)** *bdnf,* **(C)** trkb, **(D)** *nrf2*, and **(E)** *keap1* mRNA levels in zebrafish brains were assessed using real-time PCR. Each level of gene expression in GHes-fed zebrafish was relative to that in control. *n* = 8. n.s., not significant. Results are shown as means ± standard deviation.

Brain-derived neurotrophic factor (BDNF) is well known to regulate mental behavior in vertebrates. Upregulation of the BDNF/CREB pathway reportedly suppresses stress-induced anxiety behaviors (Jiang et al., 2020). In GHes-fed zebrafish, the protein level of CREB was significantly upregulated compared to that in control (3.0-fold increase, *p* < 0.05, Figure 7A and Supplementary Figure S2), accompanied by an increase in the *bdnf* mRNA level (1.5-fold increase, *p* < 0.05, Figure 7B). The mRNA expression of tropomyosin receptor kinase B (TrkB), a BDNF receptor, did not differ between the control and GH-treated groups (Figure 7C). These results suggest that downregulation of ERK/AP-1/TH1 and upregulation of the BDNF/CREB pathway may be involved in the suppression of anxiety-like behavior by GHes. Although the hypothalamic-pituitary-adrenal (HPA) axis, serotonin, and γ-Aminobutyric acid (GABA) pathways are also reportedly involved in anxiety behavior, GHes did not affect the expression of genes related to these pathways (Supplementary Figure S3).

Oxidative stress is induced by acute stimulation, resulting in neuronal damage and anxiety. *Nrf2* is a transcription factor that regulates the upregulation of anti-oxidant genes, and Keap1 interacts with *Nrf2* to inactivate it. During stress induction, reactive oxygen species (ROS) are produced, and it *Nrf2* is activated to eliminate ROS (Motohashi and Yamamoto, 2004). In the present study, GHes suppressed the mRNA level of *nrf2*, but not *keap1*, in zebrafish brains (Figures 7D,E), confirming the reduction in stress in GHes-fed zebrafish.

## 4 Discussion

**FIGURE 8:**
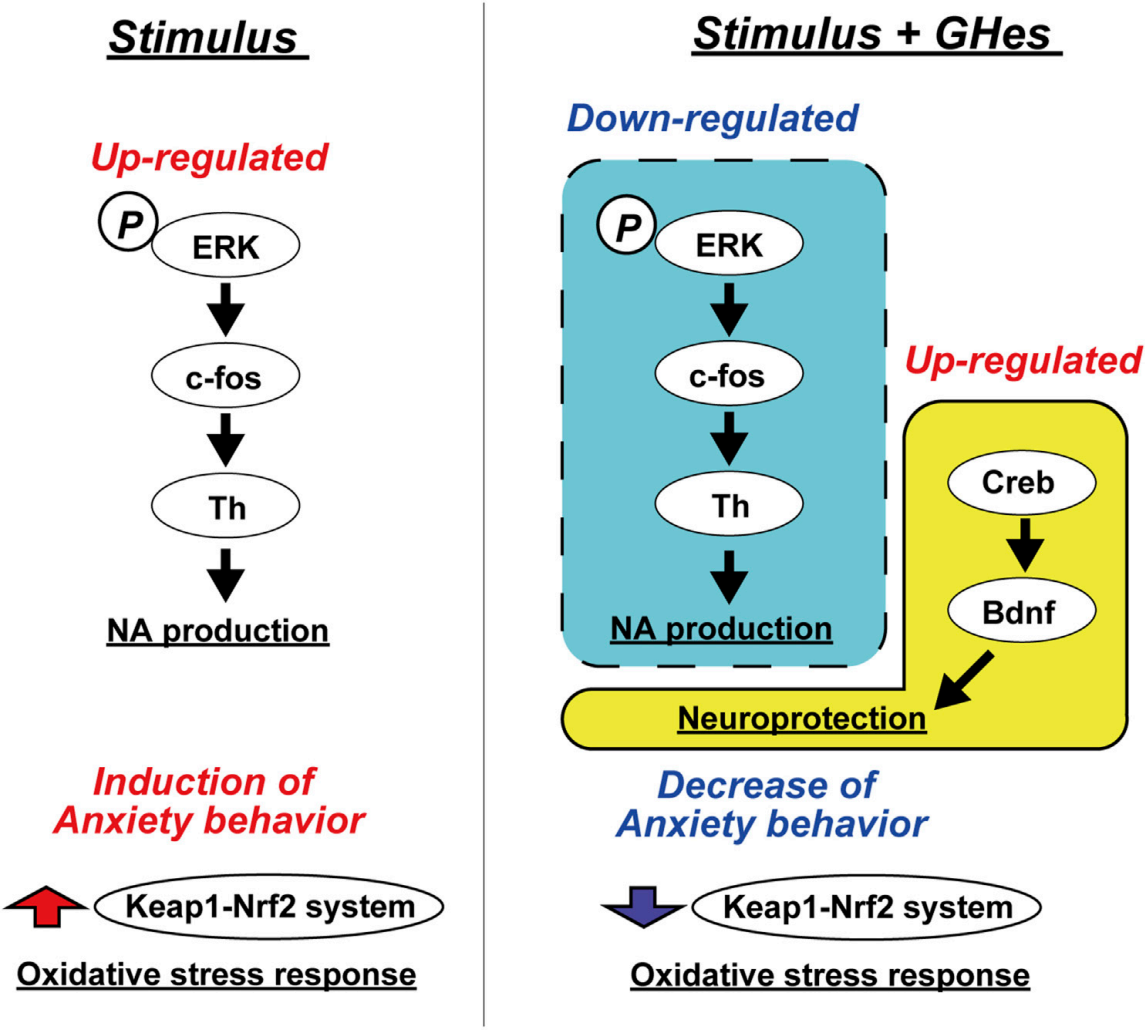
Hypothetical mechanism of the anxiolytic activity of GHes in zebrafish.

Several studies have revealed the anxiolytic and antidepressant activities of hesperidin (Hes). It is also known that hesperetin and hesperetin glucuronide, metabolites of hesperidin, can penetrate the brain through the BBB (Youdim et al., 2003). However, its low water solubility attenuates the bioavailability of Hes and metabolites. The present study evaluated the effects of glucosyl hesperidin (GHes), which improves the water solubility of hesperidin by the conjugation of monoglucose, on anxiety behavior in zebrafish induced by various stresses. GHes significantly suppressed freezing behavior in the novel tank test, low water level-induced stress test, and alarm substance test, and increased explorer activity in the white area in the black-white preference test. The anxiolytic activity of GHes was more potent than that of Hes. Furthermore, we suggest that GHes suppressed anxiety in zebrafish by attenuating the ERK/AP-1/Th1 and BDNF/CREB pathways (Figure 8).

GHes and Hes enhanced food intake in zebrafish. Hes is a significant component of the herbal medicine “Chin-pi” contained in Kampo medicine Ninjinyoeito, which is used to support the treatment of various diseases by energizing patients through improved mental health (Kawabe et al., 2021). Hesperidin may enhance feeding, and ghrelin in the gastrointestinal tract may stimulate NPY in the central nervous system, resulting in enhanced feeding (Fujitsuka et al., 2011). This is likely because feeding Ninjinyoeito to zebrafish lacking NPY did not change their food intake (Kawabe et al., 2022).

Increased noradrenaline levels are the leading cause of freezing in zebrafish under acute stress. Ninjinyoeito suppresses zebrafish freezing via the inhibition of NA neurons, and it has been shown that Chin-pi is one of the anxiolytic agents (Kawabe et al., 2021). In addition, Hes suppresses the induction of freezing in an animal model of post-traumatic stress disorder, accompanied by a decrease in noradrenaline (Lee et al., 2021). Although the inhibitory effect of Hes on freezing was also observed in the present study in several acute stress tests, the inhibitory effect of GHes on freezing was higher than that of Hes.

The BDNF/CREB pathway is deeply involved in the development of psychiatric disorders such as depression, Alzheimer’s disease, Parkinson’s disease, bipolar disorder, and memory disorders (Nagahara and Tuszynski, 2011). The CREB/BDNF pathway is involved in the mental dysregulation induced by environmental endocrine disruptors (Tang et al., 2022). Thus, this pathway has been considered a target for a drug investigation. Several compounds or extracts from natural products, such as darmmarane sapogenins originating from ginseng (Jiang et al., 2020), tannins from *Terminalia chebula* fruits (Chandrasekhar et al., 2018), and diterpene quinone Tanshinone IIA isolated from the roots of *Salvia miltiorrhiza* Bunge (Jiang et al., 2022), attenuate anxiety by activating the CREB/BDNF pathways. Flavonoids are also reported to attenuate mental disorders involved in CREB/BDNF pathway, such as rutin (Moghbelinejad et al., 2014), baicalin (Jia et al., 2021), and naringin (Gao et al., 2022). Several studies have reported the involvement of Hes in the CREB/BDNF pathway in improving memory function (Lee et al., 2022), anxiety in diabetes (Zhu et al., 2023), and pentylenetetrazole-induced convulsions (Sharma et al., 2021). The present study demonstrated the involvement of the ERK/AP-1/Th1 and BDNF/CREB pathways in GHes; similarly, glycosylation of Hes enhanced the anxiolytic activity of hesperidin itself.

The present study demonstrates the potentiation of hesperidin glycosylation for anxiolytic activity. Glycosylation of flavonoids is an effective method for enhancing their biological activity. In general, flavonoids have low solubility in water, resulting in low absorption efficiency in the small intestine. Flavonoid glycosylation improves absorption (Yamada et al., 2006). In addition, its low water solubility makes it unsuitable for food processing as a food ingredient.

Thus, this study aimed to clarify the anxiolytic effects of GHes. As anxiety is one of the leading causes of depression, its alleviation is crucial for reducing the risk of depression. The pathway through which GHes acts in zebrafish is similar to that of Hes in mammals. This indicates that GHes is a functional ingredient that enhances the action of Hes.

However, this study has several limitations. First, even though zebrafish and mice have the same metabolic system for GHes, the cranial nerves and gastrointestinal tract structures are different; therefore, an appropriate dosage and bioavailability of GHes need to be considered. Second, we examined the effects of GHes on acute stress, but the effects of GHes on chronic stress was not tested. Third, the molecular mechanisms of the action of GHes remain unclear. Recent reports have shown that Hes is involved in astrocytes (Nones et al., 2012) and that GHes could potentiate this effect. Future clarification of these limitations will lead to the practical application of the anxiolytic effects of GHes.

## Data Availability

The original contributions presented in the study are included in the article/Supplementary Material, further inquiries can be directed to the corresponding author.
