# Supplementary material for: Glucosyl hesperidin exhibits more potent anxiolytic activity than hesperidin accompanied by the attenuation of noradrenaline induction in a zebrafish model
Source: Front Pharmacol. 2023 Aug 17;14:1213252. doi: 10.3389/fphar.2023.1213252 (PMC10470464; doi:10.3389/fphar.2023.1213252)
Supplement: Supplementary file 1 [file DataSheet1.PDF]

Supplementary Material

Glucosyl hesperidin exhibits more potent anxiolytic activity than hesperidin accompanied by the attenuation of noradrenaline induction in a zebrafish model

Takumi Nishida[1], Chihoko Horita[1], Mikiya Imagawa[1], Momoka Hibarino[1], Sayaka Tateno[1], Yurina Kubo[1], Momoko Kawabe[2], Naoki Morishita[3], Shin Endo[3], Kazuhiro Shiozaki[1,2*]

[1]Department of Food Life Sciences, Faculty of Fisheries, Kagoshima University, Kagoshima, Japan

[2]Course of Biological Science and Technology, The United Graduate School of Agricultural Sciences, Kagoshima University, Kagoshima, Japan

[3]R&D Division, Hayashibara Co., Ltd., Okayama, Japan

Supplementary Table 1 Primers used in this study

Gene	Accession No	Primers		Product size (bp)
Real-time PCR				
c-fos	NM_205569.1	CAACTGTCACGGCGATCTCT	GCTGGTCAGTTTCAGCTTGC	291
keap1	NM_182864.2	GAGGGGTCACGGTTACTTGG	GGACAAACGTCTCGAAGGGT	249
nrf2	NM_182889.1	GGCGATCCTCCTGTAAACC	CGAAGGATCCGTCTTCG	172
crh	XM_009298729.3	GAATCTGCACGTGGTTGTCG	TCGTCCATGATCTTGCGGTT	384
gad1b	NM_194419.1	GCTACCAACCACAGGGAGAC	CACAACGTCGAAACATAGCCG	184
gad2	NM_001017708.2	AACTTACCGCACCAAAACCT	CCCACAACGACGCTACAATA	164
tph1a	NM_178306.3	CGAGTAAAAGCGACGGGCCA	TTCTTCGGGAACCACGGCAC	442
trkb	NM_001197161.2	GACCTCGTACTTGCCCCAAA	GGATGTCCAGGTACACAGGC	141
bdnf	NM_001308648.1	TTCTGAGCACGGCAGAAGTT	ACCTGTTGGAACATTTTCCCCTAT	132
actb	NM_131031.2	CGCCATACAGAGCAGAAGCCA	AGCACCCTGTGCTGCTCACT	938

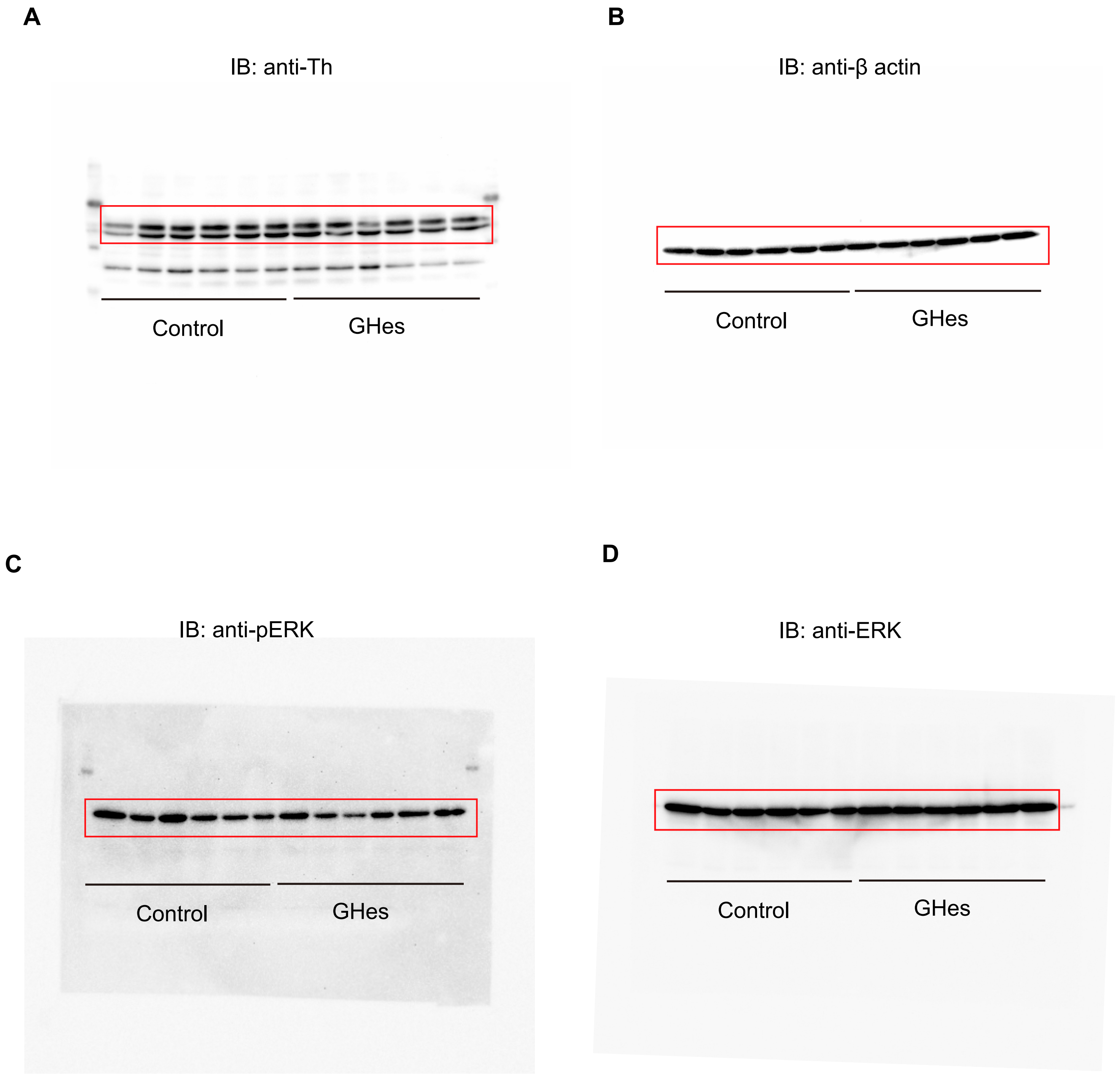

Supplementary Figure 1 Th expression and ERK phosphorylation pattern in GHes-Fed zebrafish after alarm substance treatment.

(A, B) Full length blot using anti-Th and anti-β actin shown in Fig. 6D, and (C, D) anti-pERK and anti-ERK in Fig. 6F. The regions of the original blot used in main figures are shown using red boxes.

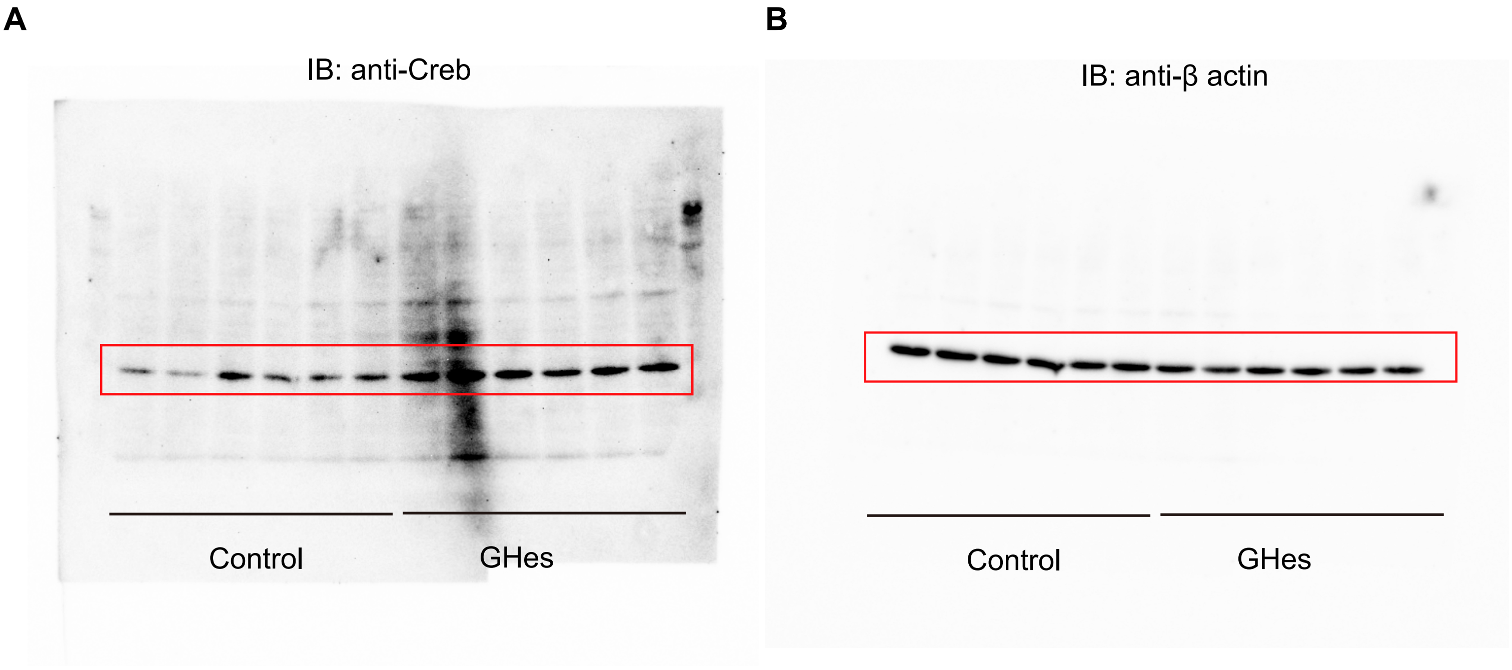

Supplementary Figure 2 Creb expression pattern in GHes-Fed zebrafish after alarm substance treatment. Full length blot using anti-Creb and anti-β actin shown in Fig. 7A. The regions of the original blot used in main figure are shown using red boxes.

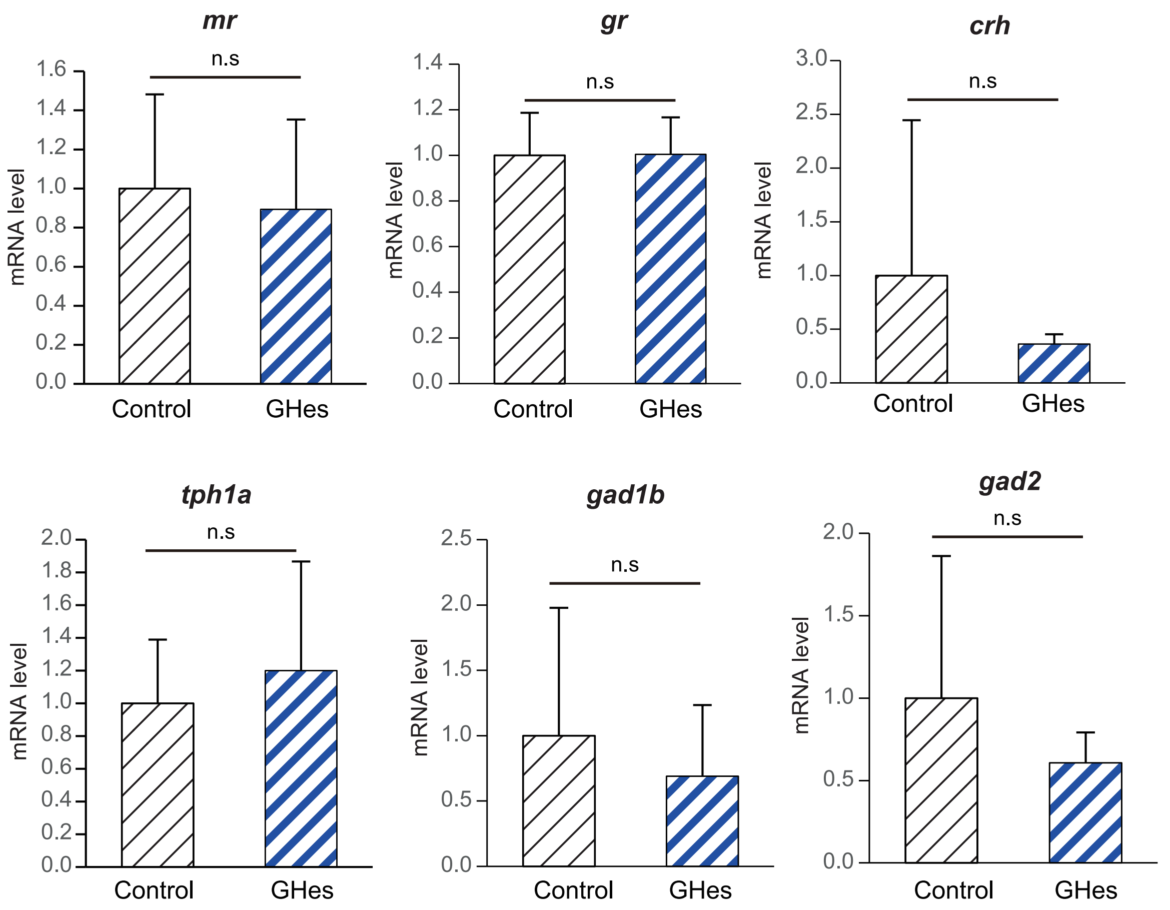

Supplementary Figure 3 Gene expressions in GHes-fed zebrafish after alarm substance treatment.

The fish brains were excised at 15 min after the alarm substance -stress. The mRNA levels of anxiety-related genes in the zebrafish brains were assessed using real-time PCR. Each level of gene expression in GHes-fed zebrafish wais relative to that in the the value in control. n = 10. n.s., not significant. Results are shown as means ± standard deviation.
